# Homeotic Evolution in the Mammalia: Diversification of Therian Axial Seriation and the Morphogenetic Basis of Human Origins

**DOI:** 10.1371/journal.pone.0001019

**Published:** 2007-10-10

**Authors:** Aaron G. Filler

**Affiliations:** 1 Department of Anthropology, Museum of Comparative Zoology, Harvard University, Cambridge, Massachusetts, United States of America; 2 Department of Neurosurgery, Institute for Spinal Disorders, Cedars Sinai Medical Center, Los Angeles, California, United States of America; Max Planck Institute for Evolutionary Anthropology, Germany

## Abstract

**Background:**

Despite the rising interest in homeotic genes, little has been known about the course and pattern of evolution of homeotic traits across the mammalian radiation. An array of emerging and diversifying homeotic gradients revealed by this study appear to generate new body plans and drive evolution at a large scale.

**Methodology/Principal Findings:**

This study identifies and evaluates a set of homeotic gradients across 250 extant and fossil mammalian species and their antecedents over a period of 220 million years. These traits are generally expressed as co-linear gradients along the body axis rather than as distinct segmental identities. Relative position or occurrence sequence vary independently and are subject to polarity reversal and mirroring. Five major gradient modification sets are identified: (1)–quantitative changes of primary segmental identity pattern that appeared at the origin of the tetrapods ; (2)–frame shift relation of costal and vertebral identity which diversifies from the time of amniote origins; (3)–duplication, mirroring, splitting and diversification of the neomorphic laminar process first commencing at the dawn of mammals; (4)–emergence of homologically variable lumbar lateral processes upon commencement of the radiation of therian mammals and ; (5)–inflexions and transpositions of the relative position of the horizontal septum of the body and the neuraxis at the emergence of various orders of therian mammals. Convergent functional changes under homeotic control include laminar articular engagement with septo-neural transposition and ventrally arrayed lumbar transverse process support systems.

**Conclusion/Significance:**

Clusters of homeotic transformations mark the emergence point of mammals in the Triassic and the radiation of therians in the Cretaceous. A cluster of homeotic changes in the Miocene hominoid *Morotopithecus* that are still seen in humans supports establishment of a new “hominiform” clade and suggests a homeotic origin for the human upright body plan.

## Introduction

At the dawn of modern genetics, William Bateson's [1] vision of the new field he had named led him to follow his exploration of Mendel with an exploration of traits underlying serially repeating elements in biology. For ninety years however, his definition of “homeotic” variation along the body axis led to little or no academic interest while the broader field he coined as “genetics” grew to dominate biology.

Among the questions that Bateson sought to address by studying homeotics was the way in which genetic change could lead to the emergence of new body plans. Neither classical morphology nor standard Darwinian analysis has provided truly satisfying explanations of such major body plan innovations as the origin of the Bilaterians by symmetric right/left replication of the organism or the origin of the vertebrates by body axis inversion of the original Bilaterian design [2]. These appear to be abrupt massively pleiotropic [3], [4] consequences of single or small number gene changes that have little to do with gradual shifts in population gene frequencies under drive from natural selection.

The discovery of the homeobox in the 1970s [5], [6], [7] and the subsequent growth of interest in developmental genetics [8], [9], [10], [11], [12], [13], [14], [15], [16] has led to a revolution in evolutionary biology. There is a new understanding of terminal addition and the emergence of a wide variety of genetic mechanisms of segmentation in the Bilateria [17], [18], [19], [20]. The recent identification of extensive similarities in the genes mediating the mechanisms of segment formation in the embryos of spiders and vertebrates [21] has further revealed the ancient nature of body axis segmental morphogenesis.

It is now reasonable to return to Bateson's project. Evolutionary change in the system of homeotic genes seems to be involved in body plan transformation. Modularity theory [22], [23] and a reexamination of mutationism in the light of modern morphogenetics [24], have opened the door to a major revision of evolutionary theory to accommodate this new understanding of body plan innovation.

Can the study of homeotic change help show how morphogenetic evolution relates to the emergence of new body plans [25], [26], [27], [28]? Do similar considerations apply to the more modest alterations in “body configuration” as it may apply to changes at the level of infraclass, order and family within the Mammalia? The advance of comparative genomics has accelerated our understanding of the way in which duplications of genes play a critical role in evolution [29], [30]. When a gene is present in a second copy, evolutionary constraints are relaxed–one copy may be altered without depriving the organism of the existing effects of the original gene. It has not been clear whether morphologies display similar patterns of change. If morphologies do evolve in this fashion, are the effects of these changes of minor or major theoretical, systematic and biological importance?

This report examines the question of whether duplications and homeotic changes have played a role in new body configuration change in three events of special biological interest-the emergence of mammals among the synapsid amniotes, the diversification of mammal groups in the Late Cretaceous, and the emergence of “hominiforms” among the catarrhine primates in the Early Miocene.

The study of axially arrayed serial homeotic characters in a group such as the mammals necessitates the study of vertebrae. This is a topic that has been relegated to limited sub-specialist and medical interest for more than 150 years. However, before Darwin, many of the major attempts to assemble a biological explanation for similarity among animals involved vertebrae explicitly. Most prominently, the widely attended zoological works of Goethe [31], [32], Geoffroy [33], [34], [35], and Owen [36] represented spinal repetition series as central to understanding biology. Recently, our new understanding of morphogenetics has triggered a new interest in this complex anatomical arena [37], [38], [39], [40], [41]. Still, the published literature on the evolutionary biology of mammalian axial structures is remarkably sparse.

In addition to the progress of axial skeletal fossil discoveries, the remarkable advances in our understanding of the embryologic development of axial structures and their relationships to *Hox*, *Pax* and other Bilaterian homeotic and morphogenetic gene families have further increased the relevance of attention to evolution of axial structures [39], [40], [42]. As we explore the hominoid genome [43], [44], we need careful analysis on where to look among the thousands of genetic differences among these species [30] to best identify critical events in the genetic genesis of human form. There is tantalizing evidence that the major changes that distinguish human vertebrae from those of Old World monkeys follow a pattern that may leave a distinct and identifiable trace in the genome.

The hominiform example is particularly compelling. Proconsulid hominoids differed from old world monkeys in having a Y-5 pattern of molar cusps but were otherwise similar to them in body form and ecological niche–most appear to have been generalized quadrupeds [45], [46], [47], [48]. A significant subsequent homeotic transformation is correlated with the emergence of novel upright (orthograde) locomotor patterns in a new hominiform clade. That makes this clade particularly interesting as a biological transformation [37], [38], [39] in addition to its importance in understanding the relationship of homeotic change to human origins.

For most of the past two hundred years, models of the origin of human upright posture and bipedalism have been based primarily on evidence from the appendicular and cranial skeleton, but evidence from the spine has played little or no role in our understanding. A series of discoveries of axial skeletal fossils from species including *Morotopithecus bishopi* [47], [49], *Proconsul nyanzae* [45], *Oreopithecus bambolii* [50], [51] and *Pierolapithecus catalaunicus* [52] have now provided evidence that is remarkably inconsistent with models that have not considered axial structures in understanding posture.

Given the many unique aspects of load bearing and movement requirements, it is not at all surprising that the lumbar vertebrae of modern humans are strikingly different in structure and function from typical mammalian vertebrae. However, the appearance of most of the unique features of the *Homo sapiens* lumbar vertebra in UMP 67-28, a hominoid fossil from 21.6 million years ago [37], [47], [49] is very surprising. This is particularly true since the apes of the Early and Middle Miocene have been generally considered to have few or none of the modifications of body plan that characterize modern apes and humans.

For a variety of reasons, the term “human” has been applied to a clade of hominoids commencing at the split from the chimpanzee lineage about six million years ago [53]. The basis for this distinction has been upright bipedalism exclusively in the human lineage. However, when the evidence from serial axial structures and homeotic events are considered, the anatomical basis for upright posture and bipedalism appears to have arisen far earlier–it is the axial anatomy first seen in *Morotopithecus*. Upright bipedalism plays a significant role in all the species of a clade that share the morphogenetic transformation with *Morotopithecus*.

The significance of the anatomical adaptations to upright posture and varying degrees of bipedalism seem among the hominoids has been a matter of ongoing interest [54], [55] [56]. Nonetheless, it has been widely accepted that specialization for full time primary bipedal locomotion did not occur in the direct human lineage until the split from chimpanzees had taken place about six million years ago.

However, when the various components of axial anatomical specialization in hominoids are fully identified, and their context in the broader setting of mammalian homeotic evolution is made clear, an alternate sequence of events becomes increasingly compelling. This is the possibility that a distinct and ancient clade within the hominoids can be identified that share a major modification of axial architecture that underlies the upright posture and primary bipedalism of modern humans. This morph appears to persist across the succeeding 21 million yeas to be preserved in primitive form in modern humans. The various other types of specialized locomotion seen among existing hominoids are made possible by comparatively minor secondary and tertiary modifications of the original primitive upright, bipedal architecture. This is the basis for asserting a homeotic transformation is the basis of the origin of humanity.

## Results and Discussion

### General Patterns of Homeotic Change in the Mammalia

**Figure 1:**
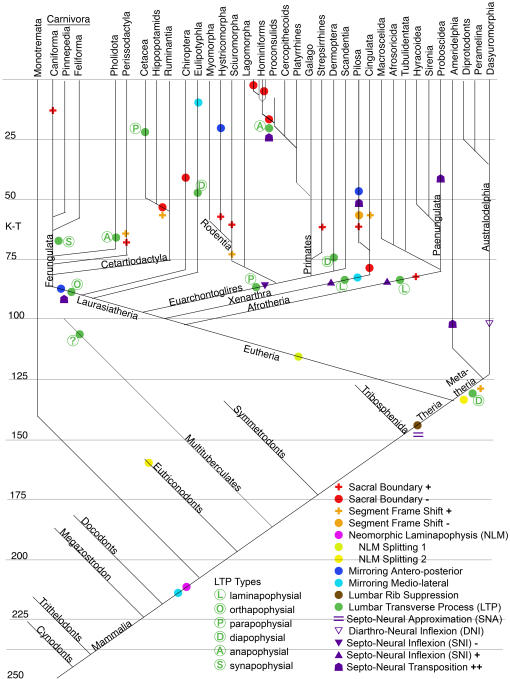
Systematic and temporal distribution of homeotic character transitions in Mammalian groups. Divergence data after Springer et al[58], Flynn et al[90], Kielan-Jaworowska et al[76]. K-T-Cretaceous-Tertiary boundary.

This study revealed that body configuration modification in the Mammalia often involves emergence and change of homeotic gradients. In a number of instances clusters of multiple different homeotic gradient changes occurred at the stem of a major systematic radiation (Figure 1).

These clusters of homeotic change generally qualify as body plan changes and often relate to significant alterations in the adaptive zone of the descendant groups. These clusters of changes are often preserved as a fixed homeotic set in the descendant group across tens of millions or hundreds of millions of years.

Within individual lineages many of the gradients demonstrate alterations on a sporadic basis (at the level of species or higher level clades). Some lineages (e.g. hominiform hominoids, pilosan xenarthrans) show a very high frequency of homeotic change for some gradients. Other lineages show little or no homeotic change over hundreds of millions of years (Monotremata).

Some homeotic alterations appear to be relatively highly conserved–they fluctuate in their expression among more ancient lineages but eventually become fixed (e.g. lumbar rib suppression). A few homeotic features never change after their initial appearance (e.g. emergence of the laminapophyis, septo-neural approximation).

At a finer level, some gradients clearly are subject to independent alteration in rate and tempo of expression along the body axis–some progress incrementally along the segmental series, some commence abruptly and then progress slowly and these properties vary across taxa. The gradients may respect medio-lateral and dorso-ventral positional relationships relative to each other or they may cross as they progress down the body axis. The segmental locations of onset of gradient change do not follow rigidly fixed sequences relative to each other.

Once established, the expression pattern of these gradients and of the morphological substrates upon which the gradients act then diversify (Figure 1). Some appear to have major functional impacts on the organism, others may have become fixed (uniformly present in descendant lineages) solely due to morphogenetic constraints.

One remarkable aspect is the mirroring and duplication of homeotic gradients. A gradient series usually seen with a given polarity and location recurs with opposite polarity at a different location. New gradients may act along the entire body axis or in replicated form within each segment. The emergence of new types of structures by duplication with subsequent diversification of the new version mimics the pattern of change often seen with gene duplication at the level of the genome.

### Segment Identity–the Primary Gradient

The basic homeotic distinction of five major spinal regions (Table 1) is apparent in the earliest land vertebrates [57] and can be assessed by boundary transitions. Seven cervical segments are standard and readily identifiable in mammals and seven to nine in most amniotes with the prominent exception of the extensive duplication and alteration of the cervico-thoracic region at the emergence of the avian winged archosaurs (birds). A very small number of mammalian species have alteration in cervical vertebral numbers on a sporadic basis.

**Table 1:** Primary Gradient–Segmental Identity and Boundaries

Feature	Category	Description	Transitions	Groups	Detail-Illustrations
Region					Figure 2, 3, 4
	Cervical				
	Thoracic				
	Lumbar				
	Sacral				
	Caudal				
Discrete boundary					
	Cervico-thoracic	First rib contacting sternum			
	Lumbo-sacral	First lateral element contacting pelvis			
			Posterior position change		Figure 2
			Anterior position change		Figure 3, 4
	Sacro-caudal				
Complex Boundary					
	Thoraco-lumbar				
		Rib head reduction			
			Loss of tubercular head		
				Euarchontoglires	Figure 2, 7B
			Shift to single capitular head		Table 2
		Diaphragmatic vertebra			
			Sagittalization of facet plane		Table 3
		Splitting of Laminapophysis			Table 3
		Elaboration of Lumbar Transverse Process			Table 4
		Septo-neural position shifts			Table 5

**Figure 2:**
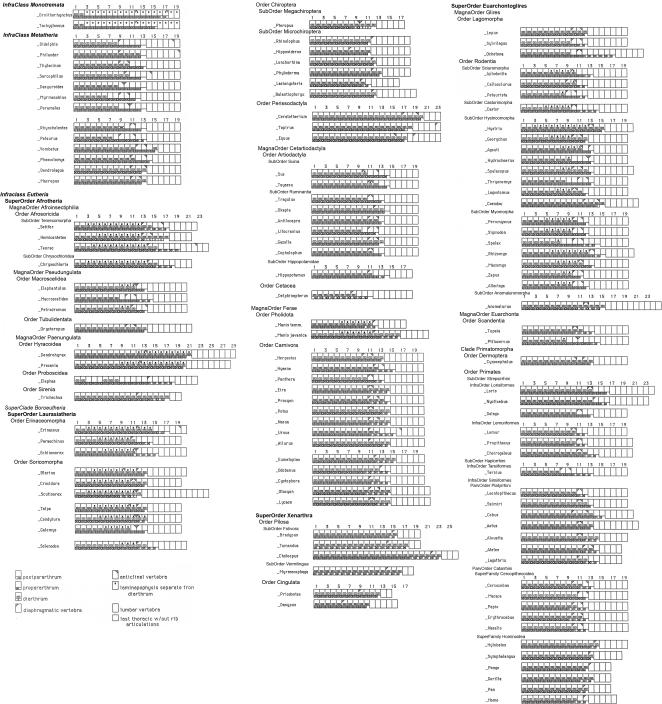
Thoracic and lumbar segmental homeotic trait patterns in mammalian species.

The thoraco-lumbar transition within the vertebral series of mammals, however, depends on a variety of gradients that defy simple counting and categorization (Table 1)–this issue is explored in detail below. The components of this transition are stably arrayed in some higher taxa but subject to frequent generation of new versions in others (Figure 2).

The lumbo-sacral boundary collectively affects multiple gradients in concert and is therefore a discreet phenomenon like the cervico-thoracic boundary. The recent advent of a molecular resolution to the deep relationship of mammalian groups [58], [59] provides an opportunity for observing phylogenetic patterns in the segmental position of the lumbar/sacral boundary. Some groups are very stable for this boundary position, some demonstrate occasional small shifts, others are quite unstable with either significant increases or decreases in number of segments (Figure 2). There are a few species with highly unusual thoraco-lumbar or lumbo-sacral boundary effects.

*Scutisorex* provides the most dramatic example of morphogenetic disruption of the homeotic system among the mammals [37] having scores or hundreds of facet pairs and a seeming duplication of the entire lumbar region. Although most mammals–including the numerous other species of the Soricidae-have six or seven lumbar vertebrae, *Scutisorex* has twelve lumbar vertebrae.

Another informative homeotic character state is the replication of the “diaphragmatic” thoraco-lumbar transition vertebra in a specimen of the macroscelid *Petrodromus tetradactylus* (USNM 241593)–a species with a remarkably accelerated rate of morphological evolution [60]. There is an elongated lamina with a double neural spine. The more posterior “third” half of the lamina replicates the anatomy of the last pre-diaphragmatic vertebra. This represents discontinuous homeotic change and shows that the joint surface reorientation seen in the diaphragmatic vertebra is indeed a homeotically determined aspect of serial morphology.

**Figure 3:**
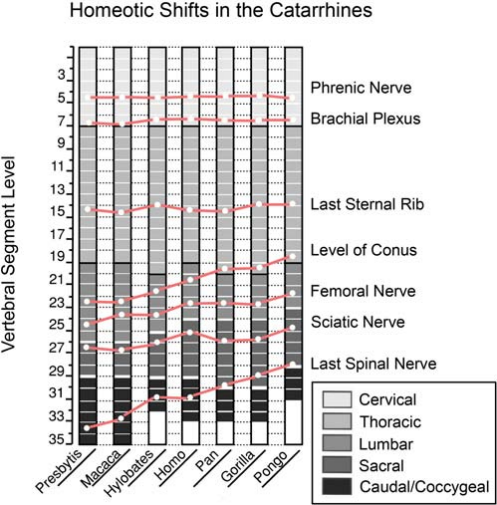
Homeotic shifts in the catarrhines. The data show the average segmental midpoint of nerve and plexus origins relative to vertebral segment regionalization (after Filler 1993 [38], some data from Keith 1902 [91]).

**Figure 4:**
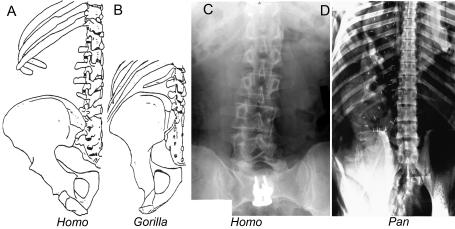
Secondary sacral boundary shifts within the hominiform clade. (A) Humans appear to retain the original hominiform longer flexible lumbar region. (B) Anatomical reconfiguration results in effective elimination of the lumbar region in *Gorilla*. (C) Despite a modal number of 5 lumbars, humans may have 4 lumbar but maintain a long flexible lumbar region. (D) Molecular phylogeny suggests that lumbar region shortening in *Pan* occurred independently and convergently (X-ray in D after Filler 1979 [92]).

Reduction in the number of dorsal (thoracic+lumbar) segments is relatively uncommon. It is typical of the Order Chiroptera and the Order Cingulata. Among hominoids this occurs in all of the species of the hominiform clade (Figure 3, 4) but not among the proconsulid hominoids. Some proconsulids may have tail loss without reduction of dorsal segment numbers [61], [62], [63] but full details of the sequence of these events remains unclear.

The initial reduction in number of lumbar vertebrae in the hominiforms appears to be a shift from the catarrhine modal number of seven down to a modal number of five or six (Figure 3). Modern humans typically have five lumbar vertebrae, the only known complete australopithecine lumbar spine has six [38], [64], [65].

Reduction to a modal number of four lumbar segments may have occurred separately in *Pongo*, then *Gorilla*, and then *Pan*, with the longer more flexible lumbar spine retained in primitive form in hominines such as *Australopithecus* and *Homo* (Figure 1,4). Alternately, the entire “great hominiform” group shared a single common secondary event causing reduction to four lumbars, but hominines subsequently reversed the trend to regain the modal fifth lumbar segment [39]. This may be consistent with the presence of upright bipedalism in the stem hominiforms, that is transformed to diagonograde postures in the common ancestor of great apes and humans, followed by rapid re-establishment of bipedalism early in the course of an independent hominine lineage.

However, as explored below, the secondary reductions of the lumbar region may be independent, parallel convergent adaptations to the various non-upright, “diagonograde” postures employed by the large apes. This interpretation, requiring an independent lumbar shortening in *Pan* after divergence from the hominines six million years ago gains some support from recent fossil evidence. *Sahelanthropus*-a candidate pre-split common ancestor of chimps and humans dated to seven million years ago-was very likely an upright biped [66], [67], [68], [69] There is also evidence for bipedalism in *Orrorin* [70] [71], [72], another hominoid dated to a period quite close to the chimp-human split. This model suggests that the upright bipedal body plan of the hominiforms arose in the Early Miocene and that since that time, there has been a continuous lineage including upright bipeds of which *Homo sapiens* is only the most recent species to demonstrate this primitive hominiform body plan.

### Frame Shifting and Rib Suppression in the Second Gradient

The two major types of segmentally repeating structures in tetrapods are ribs and vertebrae. Among mammals however, this study showed that these represent two separately determined segmental systems that may be frame shifted relative to each other. The pattern of frame shifts strongly suggests that a separate gradient for the segmental identity of ribs had emerged before the emergence of the therian group 150 million years ago.

**Figure 5:**
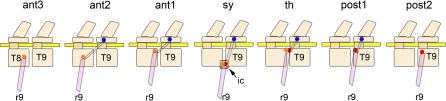
Frame shifting between rib and vertebral segments. Evidence for independent formation of a parallel segmental identity gradient for ribs that may differ from the vertebral gradient is demonstrated by frame shifting. The synapsid (sy) primitive condition has a principal (capitular) rib head articulating on a pararthrum on the intercentrum (ic) which seems to serve as a morphogenetic “target”. In basal therians (th), there is no intercentrum, but the rib head still articulates between the two centra (pleurocentra) as if the lost intercentral morphogenetic target were still present. The articulation is divided into a pre-pararthrum (red) on the anterior end of the following vertebra (iso-segmental) and a post-pararthrum (orange) on the posterior end of leading vertebra. In the posterior thorax of many eutherians (e.g. Euarchontoglires, the Xenarthran Order Pilosa) and some metatherians, the post-pararthral articulation is lost (post1)-“pre-pararthral dominance”-and the diarthral (blue) articulation is also suppressed in many groups (post2). However in metatherians, the Xenarthran Order Cingulata, Hippopotamidae and Cetacea, it is the pre-pararthrum that is lost-“post-pararthral dominance”-in the posterior thorax so that the capitulum articulates only with the post-pararthrum (ant1). The post-pararthrum may move away from the intervertebral space (ant2). In some groups, the diarthrum is also lost so that the rib (e.g. r9) articulates only with the leading vertebra (T8)-this is seen sporadically in the posterior thorax in myomorph, hystricomorph and anomaluromorph rodents and perissodactyls.

As in most tetrapods, the contact point of the rib with the vertebra has been duplicated in the dorso-ventral plane (Table 2). The more dorsally placed rib head and articulation seems to have its segmental identity determined by the original primary segmental gradient since it never demonstrates frame shifting (Figure 5).

**Table 2:** Second Gradient–Rib Head Suppression and Frame Shifting

Substrate	Category	Description	Transition	Taxa	Frequency	Illustrations
Tubercular rib head		Dorsal rib head				Figure 6A, 6C
	Articulation-single	diarthrum	articulation directly on vertebra			Figure 6A, 6C, 8B, 8C
		diapophysis	articulation on process			Figure 9C
			suppression in posterior segments			Figure 2
				Metatheria	sporadic	
				Carnivora	common	Figure 16A
				Euarchontoglires	typical	Figure 7B
				other therian groups	sporadic	
Capitular rib head		Ventral rib head				Figure 6A, 6C
	Articulation-single	pararthrum				Figure 6A
	Articulation-AP divided	post-pararthrum and pre-pararthrum				Figure 6C, 7B
			suppression in posterior segments			
				Cetacea	typical	Figure 8C
	Antero-posterior frame position					Figure 5, 6
		Intercentral	between pleurocentra			
				Early Synapsida	universal	Figure 6A1
		Post-pararthral dominance	anterior shift-progressive			
				Metatheria	typical	Figure 7C, 8A
				Cingulata	common	
				Rodentia	sporadic	
				Megachiroptera	common	
				Perissodactyla	sporadic	
				Hippopotamidae	typical	
		Antero-central (preceding segment)	anterior shift-complete			
				Cetacea	typical	Figure 8B
		Pre-pararthral dominance	posterior shift-progressive			
				Euarchontoglires	typical	Figure 7B, 12
		Postero-central (iso-segmental)				
			posterior shift-complete			
				Diapsida	typical	Figure 6A2
				Pilosa	typical	Figure 8C

Legend: Universal–all species in group; typical–sporadic exceptions; common–usual pattern with numerous exceptions; sporadic-multiple phylogenetically isolated sub-groups

Monotremes (e.g. *Ornithorhynchus*) can have mobile ribs on all of their lumbar vertebrae. In fact, many groups of Mesozoic mammals also have mobile uniarticulate ribs on their lumbar vertebrae. It is only among the therian mammals that lumbar ribs are lost definitively [73]. Since some therian mammals demonstrate suppression of the dorsal rib head (Table 2) and others demonstrate suppression of the ventral rib head (Table 2) it is not clear whether complete suppression of the lumbar ribs in therians is due to both of these traits becoming fixed in a common ancestor or due to a separate set of changes.

The more ventrally placed rib head is the principal in-line end point of the rib and is generally considered to reflect the original primitive vertebrate rib head. This rib head, and therefore the mammalian rib itself, appear to be controlled by the new, independent secondary segmental identity gradient that may be frame shifted anterior or posterior to the primary gradient (Figure 5, Table 2).

This common class of segmental ambiguities shows that numerical segment correlation between costal and vertebral elements is not a fundamental morphogenetic principal in mammals. These represent two separate seriation systems that may shift relative to each other by as much as a full segment.

### Duplications & Mirroring in the Tertiary Gradient Set

Much of the homeotic plasticity among mammals involves a number of gradients acting on a “neomorphic” or newly established structure on the dorsal (laminar) part of the vertebra that was revealed and characterized by this study. Some of these gradients appear to have profound functional significance, others seem to be best valued as windows into the morphogenetic mechanisms in play in mammalian evolution. The neomorphic element can be termed the *laminapophysis*. The evidence for either functional or morphogenetic significance comes from the widespread fixation of the character. It has been universally present in all mammalian species since it first appeared 220 million years ago.

**Figure 6:**
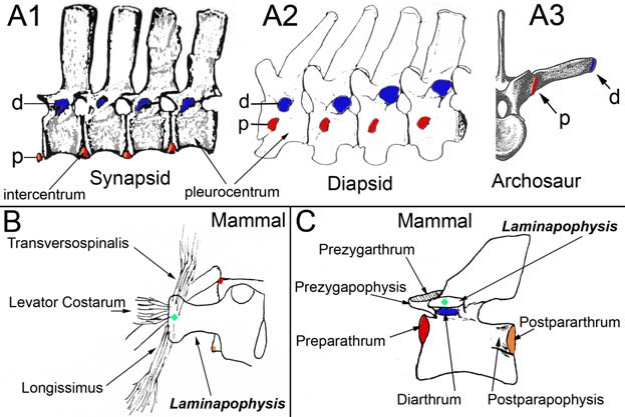
Antecedents and relations of the neomorphic laminapophysis in mammals. (A)-Configuration of diarthrum, pararthrum and intercentrum in synapsids (*Ophiacodon*) with the entire pararthrum (orange+red)) on the intercentrum (after Williston[80]) (A1), and diapsids[82] (*Crocodylus*) upper (A2) and (*Alligator*) lower (A3) thoracic. (B)-Muscle attachments of the laminapophysis. (C) New nomenclature of vertebral articular surfaces and processes in mammals. Blue-diarthrum, red-pre-pararthrum, orange-post-pararthrum, green-NLM.

**Figure 7:**
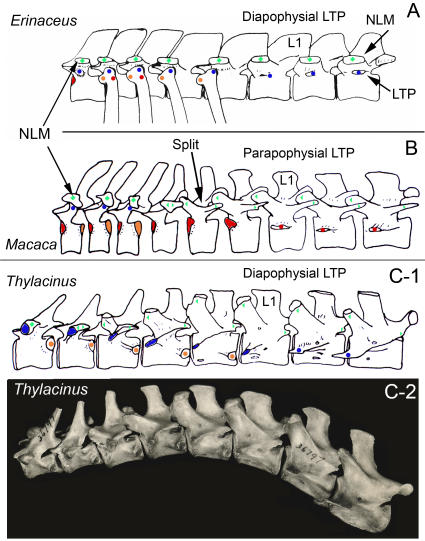
Diversity of lumbar transverse processes (LTP) serial homology and NLM morphology in therians. (A)-There is an independent laminapophysis (NLM) in *Erinaceus* (Eulipotyphla) that does not split at the thoraco-lumbar transition and is unrelated to the LTP. Erinaceomorphs have no pre-pararthrum on the last ribbed vertebra (post-pararthral dominance) and have a diapophysial LTP. (B)-Typical transition from tri-articulate rib to uni-articulate rib to LTP in Superorder Euarchontoglires. Note splitting of laminapophysis (NLM) (green), loss of the diarthrum (blue), and suppression of the post-pararthrum (orange) to yield a pre-pararthral base for parapophysial LTP (red)–drawing of *Macaca* (Primates). (C)-Post-pararthral dominance with anterior segmental frame shift in metatherians. (C1)-Diapophysial LTP with absence of prepararthrum and no participation of the post-pararthrum (orange). The last rib articulates only on the vertebra of the preceding segment. Note that the diarthrum transposes from dorsal to the neuraxis to ventral (diarthro-neural transposition). Drawing of *Thylacinus*. (C2)-Thoraco-lumbar transition in *Thylacinus cynocephalus* (Metatheria) MCZ 36797 (photo of specimen drawn in C1).

**Figure 8:**
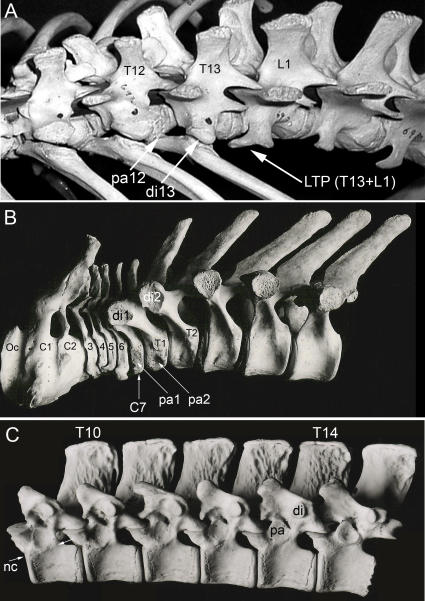
Segmental frame shifting. (A)-Anterior shift at thoraco-lumbar transition: pararthrum entirely on preceding segment with diarthrum on iso-segment. First lumbar transverse process (LTP) (on L1) is bi-segmental (T13+L1). Transitional vertebra (T13) has no capitular rib articulation and no LTP. *Macropus rufus* (Metatheria) MCZ 6930. (B)-Anterior shift at cervico-thoracic transition: pararthrum entirely on preceding segment (C7) in *Sotalia fluviatilis* (Cetacea) FMNH 99612. (C)-Posterior shift in the thoracic region: pararthrum entirely on iso-segment and migrated dorsal to the border between the neural arch and the centrum (neuro-central suture)-these two features together are analogous to the condition in archosaurian reptiles. *Myrmecophaga tridactyla* (Pilosa) FMNH 49342. Oc–occipital, C-cervical, T-thoracic, L–lumbar, di–diarthrum, pa–pararthrum, nc–neuro-central suture, LTP–lumbar transverse process.

**Figure 9:**
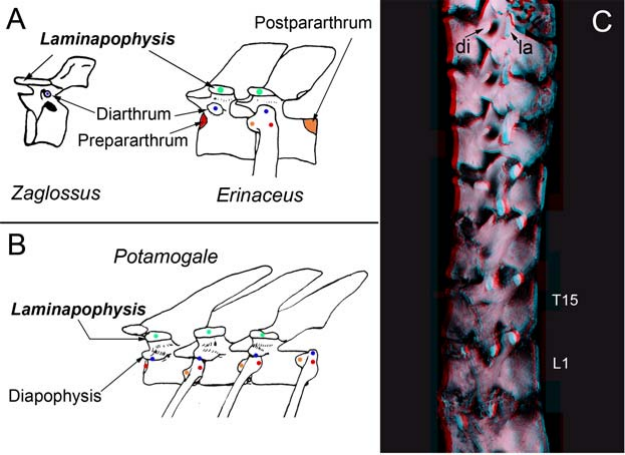
Distinction of laminapophysis from diapophysis (A)–Relation of diarthrum to laminapophysis in *Zaglossus* (Monotremata) and *Erinaceus* (Eulipotyphla). (B)–Relation of diapophysis to laminapophysis in *Potamogale* (Afrosoricida). (C)–Distinct diapophysis and laminapophysis in *Rhizomys sumatrensis (FMNH 98534)* (Rodentia). T-thoracic, L–lumbar, di–diarthrum, la–laminapophysis. Blue–diarthrum, red–pre-pararthrum, orange–post-pararthrum, green–NLM.

**Figure 10:**
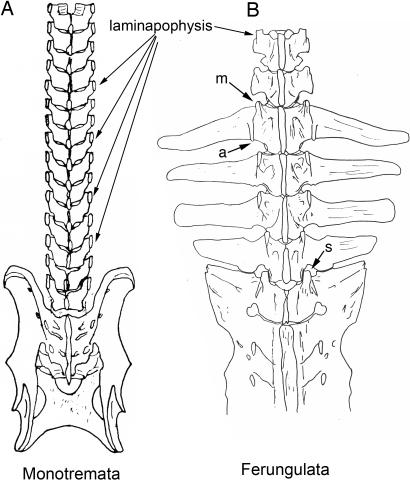
Body configuration change in mammalian axial anatomy. (A)-Monotonous laminapophysis in Monotremata (*Tachyglossus aculeata*) with no lumbar transverse process. (B)-Laminapophysis split into anteriorly directed metapophysis that slowly drifts medially to engage in sagittalization of the L4/S1 facet and posteriorly directed anapophysis. Large orthapophysial lumbar transverse processes from “third tubercle” of laminapophysial condyle on the arch (*Tapirus bairdii*, Perissodactyla). m–metapophysis, a–anapophysis, s–sagittalization.

**Figure 11:**
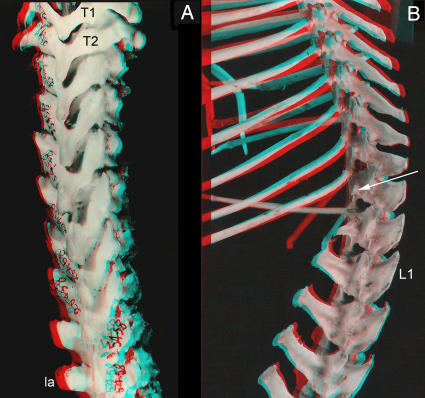
Laminapophysis and lumbar transverse processes emergence in mammals. (A)-Emergence of laminapophysis at T3 in Monotremata (*Tachyglossus aculeatus*) with no lumbar transverse processes (MCZ 25438). (B)-Emergence of orthapophysial lumbar transverse process (arrow) on vertebra also bearing a rib in small ferungulate (typical adult weight 1.5 kg) *Tragulus javanicus subrufus* (Artiodactyla) FMNH 62824. T-thoracic, L-lumbar.

The neomorph appears to arise by a medio-lateral duplication on the lamina of the vertebra. A single primitive extension or process seen in most tetrapods (the diapophysis) becomes two side by side extensions (Table 3, Figure 1, 6, 7, 8, 9, 10, 11). This effects a fundamental body configuration change in the mammalian clade.

**Table 3:** Third Gradient–Duplications and Mirroring

Substrate	Category	Description	Transitions	Groups	Illustrations
Neomorphic laminapophysis					
	Independent Status				Figure 6B, 6C, 7, 9
	Emergence	first seen at this level			
		T3		Monotremata	Figure 11A
		T1		Euarchontoglires	
		Masked	emergence hidden by mirrored structures	Xenarthra, Ferungulata	Figure 13A
	Components				
		anterior-metapophysis (mamillary)	elevates transversospinalis		Figure 6B
		middle-orthapophysis	elevates levator costarum		Figure 6B
		posterior-anapophysis (styloid)	elevates longissimus		Figure 6B, 18C
	Splitting				
			unsplit		Figure 7A, 10A
			antero-posterior separation		Figure 7B, 9C, 10B, 12
			medio-lateral separation		Figure 10B
		Polarity			
			reversal/mirroring	Xenarthra, Ferungulata	Figures 13A vs 25A
		Sagittalization			
			absent	Monotremata	Figure 10A
			articular rotation commencing at diaphragmatic joint	Eutheria	Figure 10B, 12A
			diaphragmatic joint with variable mamillary involvement	Metatheria	Figure 7C
Zygapophysis					
	Facet complex				
		Duplication	mirroring	Xenarthra	Figure 13B

Once established it actually becomes more constant than the primitive extension that it replicates. In the posterior thoracic region of many mammals, the diapophysis is suppressed along with the dorsal rib head, but the laminapophysis still appears. It is therefore clear that its morphology is determined by a new homeotic gradient that is not necessarily subject to events that alter the old homeotic gradient responsible for the diapophysis.

The laminapophysis disassociates most of the trunk musculature from the ribs, thus significantly disengaging the rib cage from the locomotor musculature of the body (Table 3, Figure 6B). This is a critical major body configuration transformation that allows mammals to progressively increase ventilation as they run at faster speeds. It establishes a “mammaliform” clade and is in many ways a defining event in mammalian origins.

**Figure 12:**
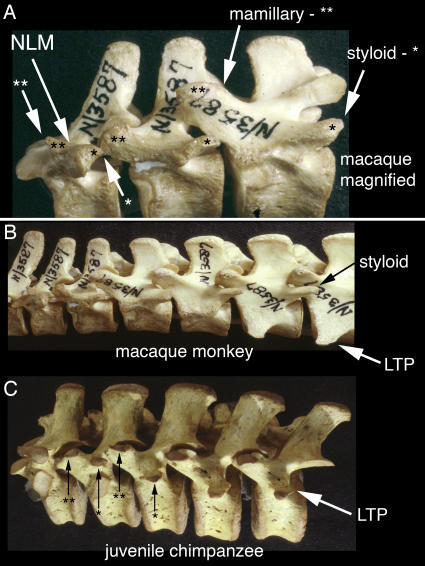
Laminapophysial splitting sequence in non-hominiform and hominiform catarrhines. (A & B)-The laminapophysis splits into anterior metapophysis (**) and posterior anapophysis (*). The anapophysis forms a posteriorly directed styloid process on the arch and does not participate in the emergence of the pre-pararthral positioned parapophysial LTP. Typical euarchontogliran style anatomy in *Macaca* (Primates) Harvard Peabody N/3587. (C)-The anapophysis (*) forms the lumbar transverse process rather than a styloid process in hominiforms (e.g. non-proconsulid apes and humans)-juvenile *Pan troglodytes*. NLM-neomorphic laminapophysis, LTP-lumbar transverse process.

At its earliest appearance there are no additional homeotic gradients affecting it. In monotremes it proceeds with monotonous uniformity of shape through all dorsal vertebrae (Figure 11A). Independently in many metatherian and eutherian mammals, however, a homeotic gradient appears to have emerged that splits it into two progressively separated halves. The extent of the split increases in more posterior segments (Table 3, Figure 7B, 12). The split also reveals an intra-segmental antero-posterior gradient replicated in each segment.

The two halves are typically also subjected to opposite medio-lateral position effects (Figure 7, 10). In eutherians the anterior half shifts medially and forces the facet to rotate 90 degrees onto its medial surface to form the “*diaphragmatic*” joint (Figure 12A). The posterior portion may shift laterally. The few therian species that do not display splitting of the laminapophysis (Figure 7A) have most likely lost it secondarily.

**Figure 13:**
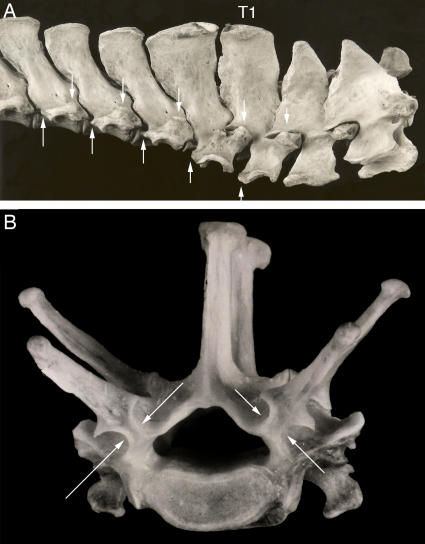
Homeotic mirroring of axial character elements. (A)–Mirrored repetition of splitting of laminapophysis into anterior metapophysis and posterior anapophysis with associated sagittalization of facet in thoraco-cervical direction in addition to the usual eutherian thoraco-lumbar gradient polarity for this sequence-*Myrmecophaga tridactyla* (Pilosa) FMNH 49338. (B)–Medio-lateral mirroring of recurved lumbar facet joints–*Dasypus novemcinctus* (Cingulata) FMNH 60493.

The most striking and widespread mirroring phenomenon among eutherians produces a separate series of “splitting of the laminapophysis” proceeding anteriorly (thoraco-cervical direction) along the spine (reverse polarity) (Figure 13A) in addition to the standard posterior progression (thoraco-lumbar direction) (Figure 7B). Most interestingly, this is associated with a mirror of the diaphragmatic joint as well. The normal one appears as part of the thoraco-lumbar transition and the mirrored one occurs at the thoraco-cervical transition.

Anterior mirroring also occurs in most carnivores, all pholidotans (pangolins), many artiodactyls and some perissodactyls suggesting that this is an echo of a single homeotic gene-based replication event in an ancient clade within the Laurasiatheria which took place after the divergence of the Chiroptera and the Eulipotyphyla (Figure 1). A similar anterior mirroring anatomy is seen in a small number of rodent species (*Hystrix, Hydrochoerus*) and almost certainly reflects an entirely independent genetic event.

Mirroring or replication of homeotic gradients also occurs in regard to several features in the Xenarthra resulting in multiple facet pairs at each articulation between lumbar vertebrae. In some species, the primary articulation takes on an unusual cylindrical shape, so the appearance of a mirror image cylinder is highly suggestive of a duplicated morphogenetic instruction (Figure 13B).

### Serial Homology of the Lumbar Transverse Process–a 4^th^ Gradient Set

**Figure 14:**
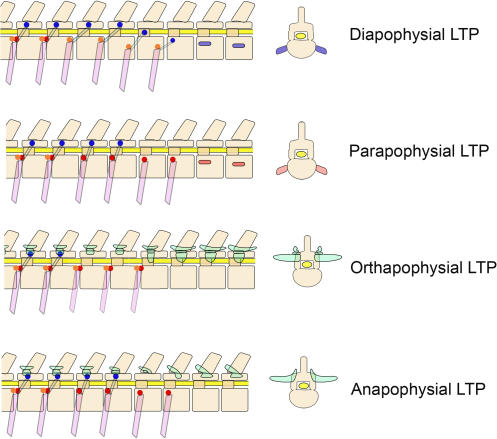
Multiple homologies for the therian lumbar transverse process (LTP). *Diapophysial LTP*: In metatherians with loss of the pre-pararthrum (red) and descent of the diarthrum (blue) onto the centrum (Figure 7C, 20), the LTP is often based on the diarthrum, occurs on the centrum, and incorporates a distal costal element as in Figure 8A. *Parapophysial LTP*: In most Euarchontoglires, the post-pararthrum (orange) and diarthrum (blue) are lost in the posterior thorax so that the LTP seriates with the pre-parapophysis and may incorporate a distal costal element as in Figure 7B. *Orthapophysial LTP*: In most ferungulates, the final rib has both a pre-pararthrum (red) and a post-pararthrum (orange) but no diarthrum (blue) as in Figure 11B. However the horizontal septum–which appears to be involved in inducing LTP formation–is dorsal to the neuraxis (see Figure 20) and the LTP is based on the middle portion of the condyle of the laminapophysis (green) (see Figure 6B). Note that the mamillary (metapophysis) and styloid (anapophysis) are still seen as in Figure 10B. The “third tubercle” of the condyle of the laminapophysis is the orthapophysis. *Anapophysial LTP*: In hominiforms, the LTP derives from the styloid portion of the laminapophysis (green) (see Figures 12C, 18B, 27A) and so carries the insertion of the longissimus muscle that occurs on the styloid on other euarchontoglirans. A similar LTP occurs in the Pholidota as in Figure 17A. Other versions of therian LTPs may involve various components from this basic set.

Mammalian groups appear to display a virtual collapse of the homology paradigm when their different types of lumbar transverse processes (LTPs) are examined in detail. More than fifteen different types of lumbar transverse process serial homology were observed (Table 4, Figure 14) and there appear to be numerous changes in homology that occur with remarkable frequency throughout the mammalian taxonomic array (Figure 1,7,8). In these events, structures that have impressive outward similarity appear to be assembled from an array of substrates with very different embryological and structural histories.

**Table 4:** Fourth Gradient–Lumbar Transverse Process (LTP) Serial Homology

Induced Element	Class/Infraclass	Superorder/Order	Category	Groups	Description	Illustrations
Lumbar Transverse Process (LTP)						Figure 14
	Synapsida	Cynodontia	Costal			
				*Thrinaxodon*	Syndesmosed	
	Monotremata		Minimal/Vestigial/absent			Figure 10A
	Non-therian-	Multituberculata	Minimal/Vestigial/absent			
				*Nemegtbaatar*	Parapophysial (?)	
	Metatherian		Costal		Diapophysial	Figure 7C, 8A
	Therian					
		Laurasiatheria				
			Costal			
				*Erinaceus*	Diapophysial	Figure 7A
				Delphinidae	Diapophysial	
				Physeteroidea	Parapophysial	
			Neolaminar			
				Artiodactyla	Orthapophysial	Figure 11B, 23B
				Perissodactyla	Synapophysial (diarthrum fused with pararthrum)	Figure 22B (*Equus*)
				Carnivora	Synapophysial	Figure 16A
				Pholidota	Anapophysial	Figure 17A
		Xenarthra	Neolaminar			
					Laminapophysial	
		Afrotheria	Neolaminar			
					Laminapophysial	
		Euarchontoglires				
			Costal		Parapophysial	Figure 7B, 12B, 18A, 18C
			Neolaminar	hominiforms	Anapophysial	Figure 18A, 18B, 19, 26, 27

The explanation appears to be a morphological field that that varies in the site of contact of its induction point upon the vertebra. The variation affects both dorso-ventral location and antero-posterior position within the segment as it can apparently coopt a variety of different axial structures to form the lumbar transverse process (LTP) depending on the location of where its induction point impacts the forming vertebra.

A few similar antecedents appear in occasional non-mammalian synapsids [74] and even in occasional species among Mesozoic mammalian groups (e.g. the Late Cretaceous *Nemegtbaatar* (Multituberculata) [75], [76]. Embryologically, the eutherian LTP (a late forming structure)–can be unrelated to the rib (a lateral part of the initial somite mesoderm) or to the thoracic diapophysis [37]. Some versions of the LTP in the Metatheria however do appear to include an attached rib. Many therian groups do not have laterally projecting LTPs (e.g. Chiroptera, some ameridelphians), nor do they occur in most groups of Mesozoic mammals.

**Figure 15:**
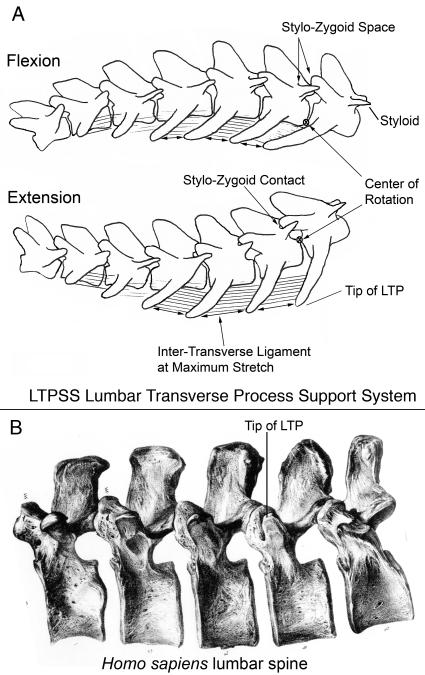
Impact of septo-neural transposition on euarchontogliran LTP suspension system in hominiforms. (A)-A convergent architecture in which LTP tips projecting ventral to the intervertebral center of rotation in most Euarchontoglirans, Carnivora, and Metatherians act to resist lumbar hyperextension by engaging and stretching elastic intertransverse ligaments. Stylo-zygoid contacts in many species further limits hyperextension. (B)-The basal hominiform architecture has LTP tips dorsal to the center of rotation and no styloids so both osseo-ligamentous mechanisms to resist gravitational hyperextension in pronograde posture are absent (after Owen 1857[93]).

**Figure 16:**
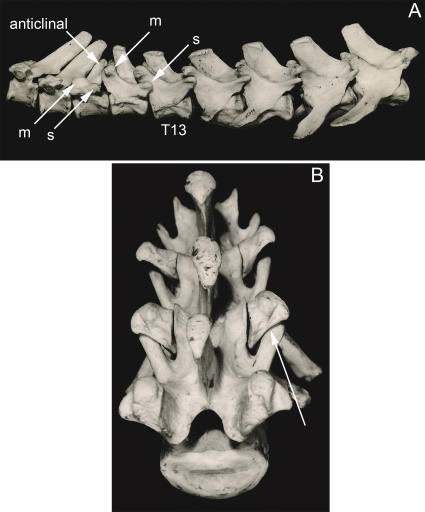
Convergent carnivoran version of LTP suspension system. (A) The LTP tips are ventral to the vertebral bodies, but they originate on the lamina as orthapophyses dorsal to the neuraxis. (B) Heavily built stylo-zygoid contacts are indicated by the arrow (*Panthera tigris* MCZ 36675). m-mamillary, s-styloid.

Large LTPs can structurally support large body size (Figure 15) [37] so parallel origins are reasonable. This body configuration innovation (Figure 11B, 16, 17B) may be part of the explanation of why limitations on mammalian body size [77] finally seem to disappear at the end of the Mesozoic.

**Figure 17:**
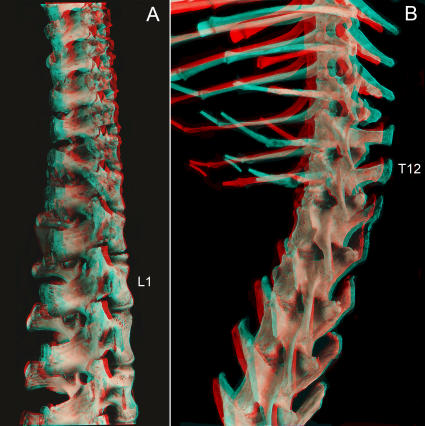
Morphological and homological lumbar transverse process (LTP) classes. (A)-The pholidotan *Manis temminckii* (FMNH 35682) has a full septo-neural transposition as in other ferungulates, but differs from the Carnivora in having purely anapophysial LTPs in place of styloid processes and maintaining the LTP tips well dorsal to the neuraxis-a set of features similar to what is seen in hominiforms. Hyperextension is limited by singly or doubly recurved cylindrical zygapophysial joints as in artiodactyls. B-The rodent *Lagostomus trichodactylus* (FMNH 53704) has the type of ventrally directed slanted LTPs seen in various ferungulate and metatherian groups-the morphology is part of the convergent LTP suspension system class, but the homology is parapophysial.

**Figure 18:**
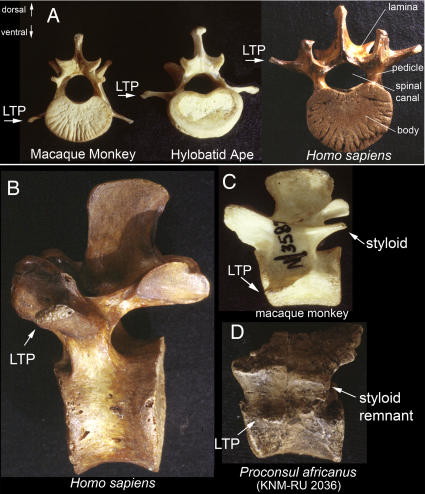
Full septo-neural transposition and styloid entrainment as anapophysial LTPs in hominiforms. (A)-The LTP (lumbar transverse process) in humans differs markedly from related primates. It is dorsal to the position of the spinal canal. It is thick and strong (triangular or box-like cross-section) instead of flat and thin. (B,C)-Styloid comparison. Lateral view of lumbar vertebrae of human, macaque monkey and *Proconsul africanus*. The human vertebra, like *Morotopithecus*, appears to demonstrate absence of the styloid process and relocation of the LTP onto the arch of the vertebra at the base of the structure that carries the facet joint. (D) The Middle Miocene proconsulid hominoid *Proconsul africanus* appears to have the more primitive LTP and styloid as seen in most euarchontoglirans.

**Figure 19:**
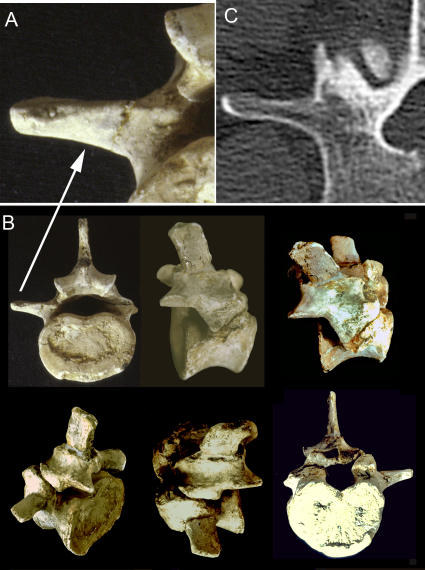
Abrupt homeotic transformation of the stem hominiform species. (A)-The lumbar vertebra of *Morotopithecus bishopi* (Early Miocene hominiform hominoid) has a shape and location of the LTP (lumbar transverse process) near the facet joint on the arch of the vertebra. (B)-The absence of a styloid process and the LTP attachment reaches above the pedicle and has the typical hominiform pattern retained in primitive form in modern humans. The pedicle is enlarged-as in humans. (C)-CT scan of modern human lumbar vertebra showing that the *Morotopithecus* LTP, pedicle, proportions and facet orientation are within the range of modern human architecture. These features suggest that *Morotopithecus* may have been the original hominiform upright biped as a consequence of a cluster of homeotic mutational events.

Cetacea display two distinct types (Table 4)–one type in the Delphinidae and the other in the Physeteroidea [37], [78]. There are also two very different types in hominoids. LTP homology distinguishes the extinct proconsulid hominoids and other catarrhines which share the typical euarchontogliran pattern from a separate clade of hominoids that share the novel and unusual LTP homology (Table 4, Figure 18, 19). Alone among the mammalian orders, the Pholidota have the same LTP homology as occurs in hominiforms [37] (Table 4, Figure 17A) although the cylindrical joints in Pholidotans result in a limited functional impact of this architecture that is quite different from the effect in the involved hominoid clade.

The transition in LTP homology is a key basis for the proposal in this paper to establish a “*hominiform*” clade within the Hominoidea. The resulting relocation of the LTP structural support is the fundamental functional change that underlies upright posture in hominiforms. This character is first seen at 21.6 million years ago in the lumbar vertebra of *Morotopithecus bishopi* [37], [49], [79] (Figure 19).

### Dorso-Ventral Transposition-a 5^th^ Gradient

Division of the chordate body into dorsal and ventral portions is defined by a rib-bearing horizontal septum in vertebrates and by dorsal and ventral divisions of the ramifying segmental spinal nerves. It is conventional to appreciate that vertebrate bilaterians have their neural tube dorsal to the horizontal septum while invertebrate bilaterians have the neural axis ventral to it. Overall, this is an issue of the fundamental patterning mechanisms of the dorso-ventral gradients of morphogenesis as well as a key point in the systematics of the Bilateria.

Oddly enough, in humans the horizontal septum is actually dorsal to the neural axis in the lumbar region. In fact, this situation occurs sporadically in groups appearing in various lineages scattered throughout the therian mammal phylogeny (Table 5; Figure 1, 17A, 18) and it is also standard in the Archosauria (Figure 6A3).

**Table 5:** Fifth Gradient–Dorso-Ventral Inflexions and Transpositions

Substrate	Category	Feature	Transitions	Description	Groups	Frequency	Illustrations
Horizontal body planes							Figure 20
	Horizontal Septum-Anterior portion						
			Ventral to neuraxis & ventral to vertebrae				
				Inferior pleurocentral position	Non-mammalian Synapsids	universal	Figure 6A1
			Adjacent to neuraxis & dorsal to vertebral centrum				
				Septo-Neural Approximation			
					Non-therian mammals	typical	Figure 9A
					Theria	universal	Figure 6C, 7A
	Horizontal Septum-Posterior portion						
		Septo-neural inflexion					
			Ventrad inflexion		Euarchontoglires	common	Figure 7B, 12B, 18A
			Dorsad inflexion		Ferungulata	common	Figure 11B
					Euarchontoglires	sporadic	Figure 25B
					Afrotheria	typical	
		Septo-neural transposition					
			Septum completely dorsal to neuraxis		Archosauria	universal	Figure 6A2/6A3
					Artiodactyla	typical	Figure 23A
					Pholidota	typical	Figure 17A
					hominiforms	typical	Figure 18A
		Septo-neural colinearity					
			Septum obstructs neural foramina	Intra-pedicular foramina	Perissodactyla	typical	Figure 22B
					Artiodactyla	sporadic	Figure 23D
	Diarthral line-posterior portion						
		Diarthro-neural transposition					
			Diarthral line becomes ventral to neuraxis		Metatherians	common	Figure 6C

**Figure 20:**
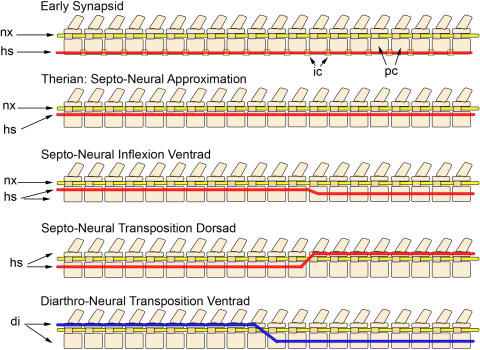
Approximation, inflexion, and transposition of horizontal body planes. *Early synapsid*: The horizontal septum (hs) incorporates the ribs and the principal rib head which articulates on the intercentrum (ic) that is in a ventral location between pleurocentra (pc). This places the septum ventral to the neuraxis (nx) and the pleurocentra (Figure 6A1). *Therian Septo-Neural Approximation*: In therian mammals, the intercentra are lost and the horizontal septum is repositioned to be just ventral to the neuraxis (Figure 7A). *Septo-Neural Inflexion Ventrad*: In most groups in the Euarchontoglires, the horizontal septum shifts ventrad away from the neuraxis in the lumbar region (Figure 12B). Dorsad inflexions occur sporadically throughout the Theria. *Septo-Neural Transposition Dorsad*: The horizontal septum is actually transposed to be dorsal to the neuraxis in hominiform hominoids (Figure 18A), the Ferungulata, many groups in the Afrotheria and Xenartha and sporadically in other groups including some rodents (Figure 25B). *Diarthro-Neural Transposition*: The diarthral plane of tubercular rib heads transposes to be ventral to the neuraxis in many australodelphian metatherians (Figure 7C).

Dorso-ventral transposition of the horizontal septum and of the neuraxis occurs at a crossing point that may be termed the “septo-neural inflexion point” and reflects the crossing of two somewhat independent morphogenetic gradients (see Figure 20).

The ancestral synapsid condition [80], is to have the horizontal septum ventral to the neural canal and ventral to the entire vertebral body (Figure 6, 20). This is the condition still seen in cynodont synapsid reptiles that are closely related to the stem mammals [74]. The mammalian condition in which the horizontal septum is moved to a position dorsal to the vertebral body is first seen in monotremes [81].

The details are still unclear for some Mesozoic mammal groups, but for all therian mammals there is a major shift of the pararthrum (and horizontal septum) to a position near the dorsal margin of the vertebral body (Figure 1, 7A). This reveals a major body configuration change that brings the horizontal septum nearly adjacent to the neuraxis. This clearly occurred in the stem therian clade around 150 million years ago and almost never varies in the thoracic region.

Embryologically and evolutionarily, the ribs arise at intersection lines between the horizontal septum and segmental myosepta. Because of this, the relatively dorsal or relatively ventral position of the attachment point of a costal derived process or lumbar transverse process on the vertebra reveals the relative position of the septal and neural horizontal body planes in the animal.

In Archosaurs, there is a very abrupt and complete inflexion and transposition (Figure 6A2/3). In the posterior neck and most anterior thorax the primary rib head is on the mid part of the vertebral body–ventral to the neuraxis. In most of the thorax, everything moves completely dorsal to the neuraxis [82].

**Figure 21:**
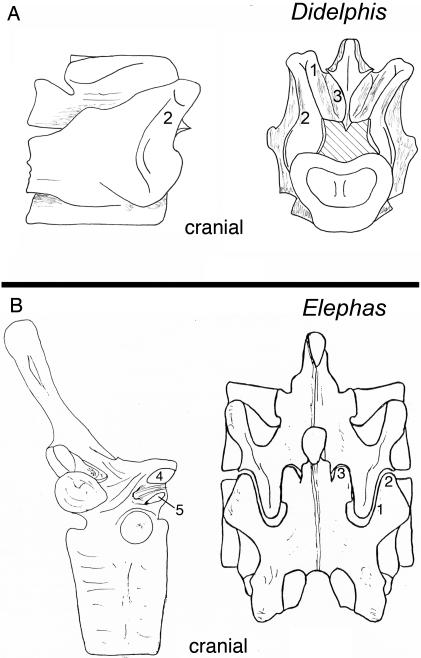
Laminar articular engagement in the setting of septo-neural transposition. (A)-Multiplication of facet surfaces for bony contact of laminar structures to resist hyperextension in the small ameridelphian marsupial *Didelphis virginianus* (MCZ 1069). (B)-Multiplication of facet surfaces with similar effect in *Elephas maximus* (MCZ 19157) (Proboscidea).

The particular type of transition seen in archosaurs almost never occurs in mammals because the synapsid/mammalian primary rib articulation tends not to cross the “neuro-central suture” of the vertebra (where the pedicle meets the vertebral body embryologically). In mammals, when the horizontal septum becomes transposed to a position dorsal to the neuraxis, there may be non-costal lumbar transverse processes (as in humans) but there are almost never ribs dorsal to neuraxis. Exceptions to this occur in the form of rib articulations on the pedicles in Superorder Xenarthra (Order Pilosa) (Figure 8C) and in the Paenungulata in Superorder Afrotheria (Figure 21B).

Septo-neural inflexion patterns have not been previously appreciated as an important aspect of tetrapod morphologic and functional evolution. Nonetheless, they may play an important role in the emergence of large cursorial mammals at the close of the Mesozoic, the emergence of the Carnivora from the ungulates at the Cretaceous-Tertiary boundary and in the origin of the anatomical basis of upright posture in humans in the stem hominiform hominoids of the Early Miocene.

A different type of change in horizontal body planes occurs in most australodelphian metatherians. This is the transposition of the ancient more dorsal rib articulation plane (diarthral plane) to become ventral to the neuraxis in the lumbar region (Table 5, Figure 7C, 20). This change reveals a separate or “third horizontal plane” within this 5^th^ gradient set that specifies the dorso-ventral position of the diarthrum relative to the neuraxis as well as its relation to the horizontal septum.

In australodelphians, there is never any further dorsal shift of the horizontal septum. Many eutherians including the Eulipotyphla in the Superorder Laurasiatheria show a similar stable relation of the horizontal septum and the neuraxis.

Dorsal repositioning of the horizontal septum is typical of the superorder Afrotheria. In proboscideans, some members of the group display a full transposition [83]. As in most ferungulates with a full transposition, paenungulates have convergent modification of their lumbar facets to rigidify the spine against extension (Figure 21B).

**Figure 22:**
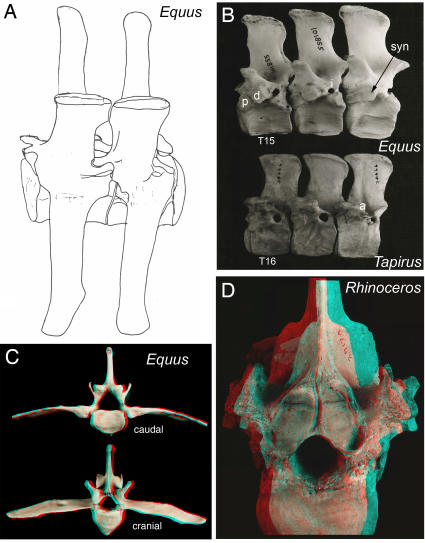
Laminar articular engagement with opisthocoely in the Perissodactyla. (A)-opisthocoelous vertebral centra-anteriorly directed ball shaped surface constrains motion so facets lock to prevent hyperextension. Supplementary facets may occur between spinous processes (*Equus caballus*). (B)-The partial ventral shift modifying an ancestral septo-neural transposition places the horizontal septum co-planar with the neuraxis so the intervertebral foramina are obliterated. The nerves exit through perforations in the pedicle. There are supplementary articulations between the successive expanded pedicles in *Equus burchelli* (FMNH 101855) and *Tapirus bairdii* (FMNH 34666). Note fusion of the pararthrum and diarthrum to form a synarthrum in *Equus*. p-pararthrum, d-diarthrum, i-intrapedicular foramen, a-anapophysis, syn-synarthrum. (C)-Opisthocoely and supplementary articulations at the base of the LTP and at the ventral margin of the vertebral body in *Equus burchelli* (FMNH 101855). (D)-Opisthocoely and biplanar pitching of the receiving facets in the rhinoceros *Ceratotherium simum* (FMNH 29174) as in other perissodactyls.

**Figure 23:**
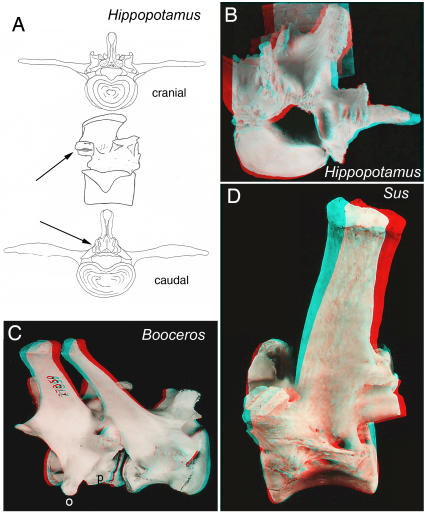
Laminar articular engagement in the Artiodactyla. (A, B)-*Hippopotamus amphibius* (FMNH 22367) demonstrating full septo-neural transposition (septum dorsal to neuraxis) and the double fluted articular system seen in many artiodactyls to block lumbar hyperextension. (C)-Single fluted locking cylinder articulation (as in pholidotans) and orthapophysial LTP [o] in *Boocercus eurycerus* (MCZ 27850) with preparthrum [p] (rib-bearing) on the same vertebra as is typical in the Artiodactyla. (D)-Double fluted articulation and separate pedicular perforations for the dorsal and ventral ramus of the exiting segmental spinal nerve in *Sus scrofa* (FMNH 92908).

Dorsal repositioning of the septum is universal in the Ferungulata (Figure 16, 17, 22, 23). Artiodactyls, Cetaceans, and Pholidotans typically have full transposition suggesting that this is the primitive condition for the Ferungulate group and preceded their diversification in the Cretaceous.

In Perissodactyls, the septum apparently undergoes a secondary and partial ventral descent. The result is the obliteration of the neural foramina since the septum and the neuraxis become co-linear. The nerve roots in perissodactyls exit the spinal canal through perforations in the pedicle and they do not have intervertebral neural foramina as in most vertebrates (Figure 22).

**Figure 24:**
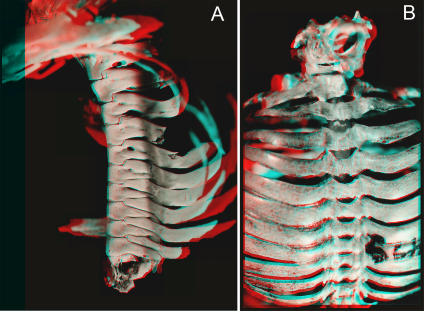
Thoracic rigidification for ventilation during flight. (A)-Arcade of interdigitating linearly extended capitular rib heads articulating with pedicles in *Rhinolophus affinis* (MCZ 56962). (B)-Costo-diapophysial fusions in *Rhinolophus ferrum* (FMNH 84499).

Some artiodactyl groups that have secondary ventral shifting of the horizontal septum also have co-linearity with the neuraxis and thus have parallel evolution of the pedicle perforations for the nerve roots instead of intervertebral foramina (Figure 23). Nerve exits through punctures in the pedicle also occur in groups with no relevant septal repositioning such as the monotremes and the Chiroptera where they relate to a dorso-ventrally expanded rib articulation that obliterates the intervertebral foramen (Figure 24).

In the eutherian Superorder Euarchontoglires, the horizontal septum is parallel to or just ventral to the neural canal (Figure 7B). However, in those euarchontoglirans with LTPs (primates, rodents, dermopterans), the septum often repositions in the opposite direction, becoming significantly ventral to the neuraxis in the lumbar region (Figure 7B, 18).

The principal exception to this in the Euarchontoglires is the case of humans and their ancestors among the hominiform hominoids. In hominiforms, there is an abrupt and strongly positive dorsal repositioning. In modern humans, for example, this relocates the septum to be completely dorsal to the neuraxis (Figure 18) and may be classed as a full septo-neural transposition. This feature is first seen in the lumbar vertebra of *Morotopithecus bishopi* dated at 21.6 million years ago (Figure 19) [37], [46], [47], [49] and reflects an extraordinarily unique reorganization of the thoraco-lumbar transition in the Superorder Euarchontoglires. This is one of the bases for the proposed identification of a hominiform clade of hominoids. This 22 million year old septo-neural transposition event has been completely preserved in modern humans and appears to be closely linked to the emergence of upright or orthograde postures in this group.

The term “human” is applied to hominoids that are upright bipeds (regardless of brain size, language, etc.) so this event may literally be the anatomic determinant of “humanity”. Although it is conventional to apply these criteria only to a “hominine” clade originating about six million years ago, the understanding of the impact of this septo-neural transposition event is a formidable challenge to that framework. If the same feature and same genetic event that underlies human upright posture and bipedalism is simply preserved in its primitive form in the stem hominines of six million years ago, how do we exclude the original species in which it appears–*Morotopithecus bishopi*?

### Joint Multiplication and Mechanical Blocks Against Extension

There is a common functional requirements of the spine in quadrupedal therians to resist hyperextension due to gravity while allowing dorso-ventral flexibility in locomotion. It is therefore not surprising that there are multiple convergent anatomical structural solutions. Most of these have not been appreciated in earlier attempts to model the mammalian spine on a global engineering basis without adequate attention to the context and detail of the specific anatomical structures actually involved [84]. This study reveals that these all tend to involve the neomorphic laminapophysis and LTP gradients. Both of these structures demonstrate a high degree of morphogenetic plasticity in therians. The participation of these structures in serial/homeotic control systems may also play a roll in their tendency to be deployed as the bases for convergent novel structures.

Universally in the Ferrungulata, Paenungulata, Xenarthra and Ameridelphia where septo-neural transposition takes place, there are supplementary modifications of the lumbar spine that relate to resistance against extension of the spine (Table 6). Typically, these involve modifications to provide rigid bony resistance to lumbar hyperextension either through elaboration of multiple additional joint surfaces (Figure 21, 25A) and/or mechanical locking systems (Figure 22, 23). These changes commence in the fossil record after the appearance of splitting of the laminapophysis 130 million years ago and appear to be modifications on a theme based on morphologic modification of the laminapophysis.

**Figure 25:**
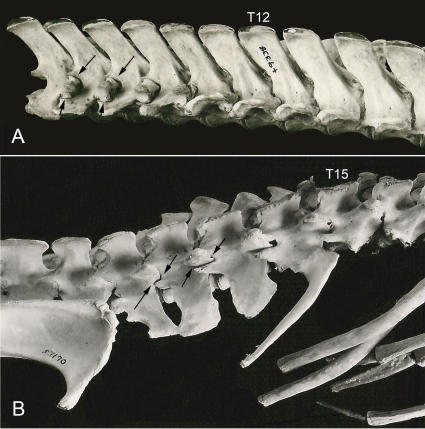
Supplementary facets. (A)-*Myrmecophaga tridactyla* (FMNH 49338) (Pilosa, Xenarthra) demonstrating extra lumbar articulations that seem to appear as a consequence of a morphogenetic replication. (B)-Supplementary facets forming at contact points between the medial styloid and the lateral mamillary processes in *Hystrix cristata* (FMNH 57170) one of the few rodent groups to demonstrate septo-neural transposition.

**Table 6:** Functional Pattern 1–Dorsal Compressive

Function	Category	Description	Transitions	Groups	Illustrations
Resistance to Extension					
	Dorsal Compressive				
		Facet multiplication			
			Zygarthral duplication		
				Metatheria	Figure 21A
				Xenarthra	Figure 13B, 25A
				Afrotheria	Figure 21B
			Mamillary-Styloid Joints (MSLM metanarthra)		
				Carnivora	Figure 16B
				Euarchontoglires	Figure 25B
				Afrotheria	Figure 21B
		Laminar articular engagement			
			Opisthocoely with blocking facets	Perissodactyls	Figure 22
			Cylindrical facets	Artiodactyla	Figure 23B
				Pholidota	Figure 17A
				Xenarthra	Figure 13B
			Double Fluted facets		
				Artiodactyla	Figure 23A, 23C, 23D

Multiplication of joints in the Superorder Euarchontoglires always involves new surfaces on the styloid process but is limited to a small number of groups including the large rodent *Hystrix cristata* (porcupine with weight up to 30 kg–and note much larger extinct related species such as *Neosteiromys pattoni*) (Figure 25B). Among primates, this occurs in some prosimians. This feature also occurs in the Afrotheria where it is seen in both the Proboscidea and in the afrosoricid insectivore *Tenrec*.

### Convergent Ventrally Tensioned LTP Arrays

In a number of mammalian groups including both therians and metatherians [85], the lumbar transverse processes display a striking slanted array that is angled ventrally so that the tips are well below the ventral margin of the vertebral bodies (Table 7; Figure 11B, 12B, 16A, 17B). The underlying serial homology is unique in each group but the functional anatomy is obviously highly convergent and independently evolved in parallel.

**Table 7:** Functional Pattern 2–Ventral Tensioning

Function	Category	Description	Versions	Groups	Illustrations
Resistance to Extension					
	Ventral Tensioning	Lumbar transverse process support system with diagonal array			
			Parapophysial		
				Euarchontoglires	Figure 15A, 17B
			Orthapophysial		
				Artiodactyla	Figure 11B
			Synapophsial		
				Carnivora	Figure 16A
			Diapophysial		
				Metatheria	Figure 7C, 8A

These and other types of arrays with the tip of the LTP ventral to the effective axis of rotation for lumbar extension participate in a dynamic, elastic, ligamentous system that supports the lumbar region and resists extension (Figure 15). This appears to be an almost opposite architectural solution by comparison with the rigid bony locking systems that also act against extension (described in the previous section).

In this elastic system, as the vertebral column passes into extension, LTPs whose tips are below the effective intervertebral axis of rotation begin to separate from each other (Figure 15A). This applies tension to the heavy, elastic intertransverse ligaments. These systems are more common in groups that do not have transposition of the horizontal septum.

**Figure 26:**
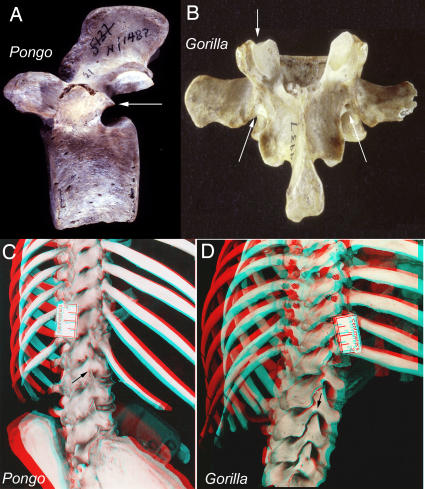
Laminar articular engagement in great apes-*Pongo* facet locks and *Gorilla* laminar blocks. (A)-Lateral view of orangutan lumbar vertebra: the inferior facet is close to the pedicle (compare with human configuration in Figure 18) and a locking extension assures hard bone to bone contact with the superior facet of the next lower vertebra *Pongo pygmaeus* Harvard Peabody N/1482. (B)-Dorsal view of gorilla vertebra showing the groove on the superior facet and notch in the lamina that that limit extension (*Gorilla gorilla* Harvard Peabody 9937). (C)-Developing facet lock in juvenile orangutan (*Pongo pygmaeus*, juvenile, FMNH 53203). (D)-Developing facet block in juvenile gorilla (*Gorilla gorilla*, juvenile FMNH 18398).

One group in the Euarchontoglires with septo-neural transposition is the hominiform hominoids. However only *Pongo* and *Gorilla* have bony blocks to lumbar hyperextension that mimic the situation in ungulates (Table 8; Figure 26). These features are seen in young juveniles and are not degenerative [37] (Figure 26). This type of block to extension is engaged when these species locomote on all fours in a diagonograde posture (body carried at about 45 degrees rather than upright orthograde or horizontal pronograde).

**Table 8:** Functional Pattern 3–Dorsal Tensioning

Function	Category	Description	Versions	Groups	Illustrations
Resistance to flexion only					
	Reversed mechanics	No ligamentous or osseous resistance to extension		Bipedal orthograde hominiforms	Figure 15B, 18A, 28A/B/C
Specialized resistance to extension					
	Secondary extension restriction	Secondary modification in context of hominiform architecture		Diagonograde hominiforms	
			Pedicle facet locks	*Pongo*	Figure 26A, 26C
			Laminar facet block	*Gorilla*	Figure 26B, 26C
			Ilio-lumbar suspension	*Pan*	Figure 28D

**Figure 27:**
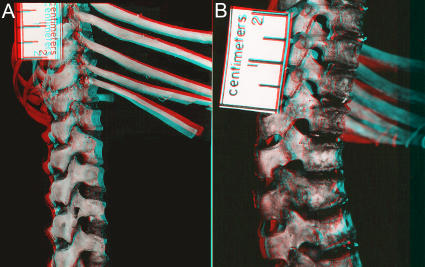
Anapophysial serial homology of the LTP in hylobatids. (A)-L1 showing seriation of styloid portion of split laminapophysis to the LTP in juvenile *Hylobates cinereus* (FMNH 33543). (B)-Transition of split LTP with styloid seriating into the LTP in juvenile *Symphalangus syndactylus* (FMNH 122725).

In hylobatids, which engage primarily in suspensory orthograde locomotion and posture, there apparently is a secondary ventral shift of the septum so that the transposition is lost. Molecular evidence suggests that hylobatid divergence took place up two to three million years after the transposition event seen in *Morotopithecus*. Developmentally, juvenile specimens of *Symphalangus* and *Hylobates* demonstrate the unusual LTP that is typical in hominiforms (Figure 27)-this shifts into a more ventral position as the individual matures.

**Figure 28:**
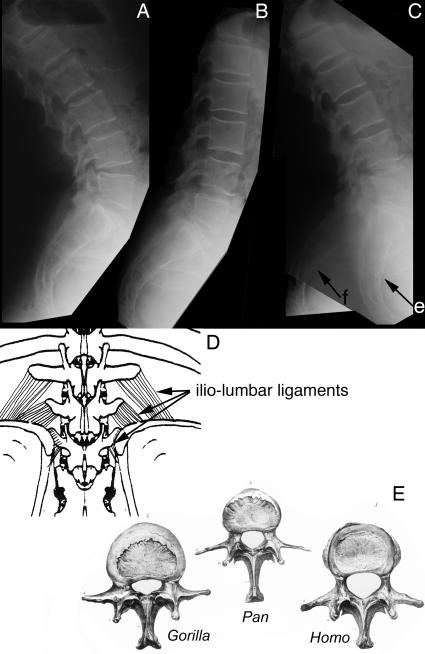
Lumbar extension in *Homo* and *Pan.* (A)-Lumbar extension in human with six lumbar vertebrae. (B)-lumbar flexion in same individual. (C)-Superimposed images in flexion and extension showing that even with six lumbars, most extension takes place between L4 and the sacrum in humans. (D)-Short lumbar spine with heavy iliolumbar ligaments in *Pan* obliterating lumbar extension thereby accomplishing support for diagonograde postures. (E)-Comparison of lumbar vertebrae in *Gorilla*, *Pan*, and *Homo* (Owen 1857 [93]) showing the thin flat LTP's typical in *Pan* because of the primacy of ligament suspension under tension for LTP function rather than muscular force transmission as in other hominoids.

Unlike the situation in *Pongo* and *Gorilla*, diagonograde progression (partially horizontal body posture) in *Pan* is not supported by bony rigidification of the lumbar region. However, *Pan* differs from other hominiforms such as *Morotopithecus* and *Homo* in having thin flat lumbar transverse processes held under tension by heavy ilio-lumbar ligaments suspended between high iliac crests (Figure 28). Homeotic reduction of the lumbar region in *Pan* plays some role in preventing extension as well (Figure 3, 4).

Alone among the therian mammals demonstrating septo-neural transposition, humans have no bony or ligamentous limitation of lumbar extension (Figure 15, 18, 28). Absence of the styloid also removes the potential for the sort of stylo-zygoid restriction seen in some other therians (Figure 15A, 16B, 25B) as well. A triangular or boxlike cross section of the LTP in *Morotopithecus* and *Homo* reflects powerful dynamic application of longissimus lumborum muscular force in bipedal orthogrady as opposed to action as a passive strut in a ligamentous system more typical of suspensory orthogrady [86], [87]. Transposition, absence of limitation to extension and preservation of a long flexible lumbar region are a unique human configuration that relates to the uniquely habitual upright bipedalism seen in our species and lineage.

Since the full anatomical array of these changes in the lumbar region are seen in *Morotopithecus bishopi* in the Early Miocene (Figure 19), that stem hominiform species demonstrates what appears to be the spinal configuration of an upright biped as well. Similar configurations are now known from *Oreopithecus*, another Miocene hominoid that appear to have been bipedal and to have five lumbar vertebrae [50], [51].

Many of the features attributed here to the hominiform pattern of lumbar vertebral architecture do occur more or less sporadically in other mammalian superorders although they are rare in the Superorder Euarchontoglires and are not seen in any non-hominiform primate group. It is worth considering that each of the hominiform lineages could have undergone the septo-neural transposition and consequent loss of the styloid and the ventrally tensioned LTP array on a homoplastic basis. However, this is no more convincing than the more parsimonious suggestion that all the hominiforms known to display these features (*Morotopithecus*, hylobatids, *Oreopithecus*, *Pieralopithecus*, *Pongo*, *Gorilla*, *Pan*, *Australopithecus*, and *Homo*) share them because they emerged in a common hominiform ancestor and are preserved as a synapomorphic character set of the group.

### Conclusion

Homeotic and dorso-ventral pattern change play a significant role in the generation of new body plans among the mammals. Clusters of morphogenetic changes in stem groups at the origin of the Ferungulata, the Metatheria, the hominiform hominoids, and other superordinal and ordinal groupings have been accompanied by sets of homeotic changes that result in new body plans. Evaluating a full array of homeotic changes rather than attending to simple counts of vertebral numbers makes this pattern evident.

Homeotic change can have major adaptive effects. When a diverse radiation of taxa shares the homeotic innovations of the stem group, there is a *prima facie* case to be made that the radiation became possible because of the homeotic innovation. The duplication that generated the laminapophysis altered the synapsid body configuration to allow for increasing ventilation with rapid running at the Triassic dawn of the mammalian clade. Emergence of the lumbar transverse process (LTP) provided the basis for large body size in therians of the Late Cretaceous. Changes in three homeotic gradient systems (sacral boundary change, septo-neural transposition, and emergence of a novel LTP structure) mark the Early Miocene establishment of a body plan committed to upright postures in the hominiform hominoids.

The hominiform homeotic transformation is bracketed between the cercopithecoid/hominoid divergence around 24 million years ago and the appearance of *Morotopithecus* at 22 million years (hominiform/proconsulid divergence) so it is clear that these changes happened with some temporal proximity to each other if not simultaneously. Future discoveries from the fossil record of this time period will no doubt reveal further details about the sequence and tempo at which this body plan generating event took place.

Duplication of homeotically determined structures and gradients in the Theria clearly relate to a remarkable explosion of new mammalian body plans. Based on divergence patterns, there is considerable evidence that this took place during the ten to fifteen million years prior to the Cretaceous-Tertiary Boundary and not after it.

This is an excellent candidate explanation for the odd pattern of total absence in the fossil record of any mammals much larger than one or two kilograms for the first 160 million years of the existence of this group [77] followed by an explosion of diverse large body mammalian forms in the last 60 million years. Homeotic change that led to diminutive lumbar ribs in the early mammals increased lumbar flexibility for sagittal excursion during symmetric gaits, but seems to have left these groups without the mechanical support in the lumbar region required for the architecture of a large animal.

Therian mammals deploy symmetrical gaits for rapid locomotion [88] [89] and they employ spinal flexion and extension to do so. The principal locomotor drive is a simultaneous thrust with both hind limbs while the forelimbs are free of ground contact. This appears to be a fundamental locomotor innovation of therian mammals, but may well have appeared in correlation with the transition from the large robust lumbar costal processes in the cynodonts to the small vestigial lumbar ribs seen in many Mesozoic mammals groups.

The appearance of large mammals at the close of the Cretaceous is at least coincident with the appearance of two major types of architectural transformation of the lumbar spine to provide non-muscular support against extension in the lumbar region. These are convergent class of rigid locking systems in groups with septo-neural transposition (Figure 21, 22, 23, 25, and 26) and the convergent class of ventrally arrayed and elastically tensioned LTPs in a several other groups (Figure 15A, 16, 17B, 18C)–both classes of anatomical change based on homeotic seriation of neomorphic or duplicated structures not present in the stem mammals. In any case, there appears to have been a dramatic increase in the rate of generation of new axial body plans once diversification and duplication of homeotic gradients affecting the laminapophysis and its derivatives that commenced in the therians.

These data also support the concept of a threshold effect in diversification of the mammals–progress awaited morphogenetic innovation. This supports an enlarged role for a mutational view [24] of evolutionary drive to update classic Darwinian and New Synthesis models of the past two centuries.

## Methods

A comparative evaluation of serially repeating structures and homeotic patterning in 250 extant mammalian species and fossil forms was carried out. For extant forms, specimens in the collections of the Harvard University Museum of Comparative Zoology (MCZ), Harvard Peabody Museum, Smithsonian Museum (USNM) and Chicago Field Museum (FMNH) were selected to provide coverage of all mammalian families except for the order Chiroptera and Rodentia where coverage was at the level of the superfamily. Specimens were selected based on preparations in which a complete naturally articulated spine in which all details could be observed. The objective was to obtain a representative overview across the Class Mammalia but variation within species was not addressed extensively. In essence there simply is not sufficient material available to provide any real comprehensive assessment of variation if the full systematic array of mammals is to be covered. In addition the vertebral nomenclature of Owen [36] was updated to distinguish between processes (“apophysis” and joints “arthrum”) to clarify situation where one type of joint appears on a different type of process [37]. Two different structures on the same side of a vertebra could not have the same name.

## References

[1] Bateson W (1894). Materials for the Study of Variation Treated with Especial Regard to Discontinuity in the Origin of Species..

[2] De Robertis EM, Sasai Y (1996). A common plan for dorsoventral patterning in Bilateria.. Nature.

[3] Theissen G (2004). Developmental genetics: bittersweet evolution.. Nature.

[4] Theissen G (2006). The proper place of hopeful monsters in evolutionary biology.. Theory Biosci.

[5] Lewis EB (1978). A gene complex controlling segmentation in *Drosophila*.. Nature.

[6] Nusslein-Volhard C (1979). Maternal effect mutations that alter the spatial coordinates of the embryo of *Drosophila melanogaster*.. Symp Soc Dev Biol.

[7] Nusslein-Volhard C, Wieschaus E (1980). Mutations affecting segment number and polarity in *Drosophila*.. Nature.

[8] Kuratani S (2005). Craniofacial development and the evolution of the vertebrates: the old problems on a new background.. Zoolog Sci.

[9] Burke AC (2000). *Hox* genes and the global patterning of the somitic mesoderm.. Curr Top Dev Biol.

[10] Burke AC, Nelson CE, Morgan BA, Tabin C (1995). *Hox* genes and the evolution of vertebrate axial morphology.. Development.

[11] Nusslein-Volhard C (1994). Of flies and fishes.. Science.

[12] van Eeden FJ, Granato M, Schach U, Brand M, Furutani-Seiki M (1996). Mutations affecting somite formation and patterning in the zebrafish, *Danio rerio*.. Development.

[13] Nusslein-Volhard C (1996). Gradients that organize embryo development.. Sci Am.

[14] Carroll SB (1995). Homeotic genes and the evolution of arthropods and chordates.. Nature.

[15] Rokas A, Kruger D, Carroll SB (2005). Animal evolution and the molecular signature of radiations compressed in time.. Science.

[16] Lovejoy CO, McCollum MA, Reno PL, Rosenman BA (2003). Developmental biology and human evolution.. Annual Review of Anthropology.

[17] Jacobs DK, Hughes NC, Fitz-Gibbon ST, Winchell CJ (2005). Terminal addition, the Cambrian radiation and the Phanerozoic evolution of bilaterian form.. Evol Dev.

[18] Minelli A, Fusco G (2004). Evo-devo perspectives on segmentation: model organisms, and beyond.. Trends Ecol Evol.

[19] Minelli A, Fusco G (2005). Conserved versus innovative features in animal body organization.. J Exp Zoolog B Mol Dev Evol.

[20] Yu JK, Satou Y, Holland ND, Shin IT, Kohara Y (2007). Axial patterning in cephalochordates and the evolution of the organizer.. Nature.

[21] Stollewerk A, Schoppmeier M, Damen WG (2003). Involvement of *Notch* and *Delta* genes in spider segmentation.. Nature.

[22] Gould SJ (2002). The Structure of Evolutionary Theory..

[23] Schlosser G, Wagner GP (2004). Modularity in Development and Evolution..

[24] Stoltzfus A (2006). Mutationism and the dual causation of evolutionary change.. Evol Dev.

[25] Budd GE (2006). On the origin and evolution of major morphological characters.. Biol Rev Camb Philos Soc.

[26] Daeschler EB, Shubin NH, Jenkins FA (2006). A Devonian tetrapod-like fish and the evolution of the tetrapod body plan.. Nature.

[27] Dahn RD, Davis MC, Pappano WN, Shubin NH (2007). *Sonic hedgehog* function in chondrichthyan fins and the evolution of appendage patterning.. Nature.

[28] Davidson EH, Erwin DH (2006). Gene regulatory networks and the evolution of animal body plans.. Science.

[29] Geisler R, Rauch GJ, Geiger-Rudolph S, Albrecht A, van Bebber F (2007). Large-scale mapping of mutations affecting zebrafish development.. BMC Genomics.

[30] Carroll SB (2003). Genetics and the making of *Homo sapiens*.. Nature.

[31] Richards RJ (2002). The Romantic Conception of Life : Science and Philosophy in the Age of Goethe..

[32] Goethe JWv (1817). Zur Naturwissenschaft überhaupt, besonders zur Morphologie.. Stuttgard; Tübingen.

[33] Appel TA (1987). Cuvier-Geoffroy Debate: French Biology in the Decades before Darwin..

[34] Geoffroy Saint-Hilaire E (1830). Principes de philosophie zoologique, discutés en mars 1830, au sein de l'Académie royale des sciences..

[35] Geoffroy Saint-Hilaire E (1822). Considérations générales sur la vertèbre.. Mem Mus Hist Nat.

[36] Owen R (1848). On the Archetype and Homologies of the Vertebrate Skeleton..

[37] Filler AG (1986). Axial Character Seriation in Mammals: An Historical and Morphological Exploration of the Origin, Development, Use and Current Collapse of the Homology Paradigm-PhD Thesis..

[38] Filler AG, Doty JR, Rengachary SS (1993). Evolution of the sacrum in hominoids.. Surgical Disorders of the Sacrum.

[39] Pilbeam D (2004). The anthropoid postcranial axial skeleton: comments on development, variation, and evolution.. J Exp Zoolog B Mol Dev Evol.

[40] Narita Y, Kuratani S (2005). Evolution of the vertebral formulae in mammals: a perspective on developmental constraints.. J Exp Zoolog B Mol Dev Evol.

[41] Buchholtz EA (2007). Modular evolution of the Cetacean vertebral column.. Evolution & Development.

[42] Boszczyk BM, Boszczyk AA, Putz R (2001). Comparative and functional anatomy of the mammalian lumbar spine.. Anat Rec.

[43] Cheng Z, Ventura M, She X, Khaitovich P, Graves T (2005). A genome-wide comparison of recent chimpanzee and human segmental duplications.. Nature.

[44] Mikkelson TS (2005). Initial sequence of the chimpanzee genome and comparison with the human genome.. Nature.

[45] Ward CV, Walker A, Teaford MF, Odhiambo I (1993). Partial skeleton of *Proconsul nyanzae* from Mfangano Island, Kenya.. Am J Phys Anthropol.

[46] Young NM, MacLatchy L (2004). The phylogenetic position of *Morotopithecus*.. J Hum Evol.

[47] MacLatchy L (2004). The oldest ape.. Evol Anthropol.

[48] MacLatchy L, Gebo D, Kityo R, Pilbeam D (2000). Postcranial functional morphology of *Morotopithecus bishopi*, with implications for the evolution of modern ape locomotion.. J Hum Evol.

[49] Walker A, Rose MD (1968). Fossil hominoid vertebra from the Miocene of Uganda.. Nature.

[50] Kohler M, Moya-Sola S (1997). Ape-like or hominid-like? The positional behavior of *Oreopithecus bambolii* reconsidered.. Proc Natl Acad Sci U S A.

[51] Rook L, Bondioli L, Kohler M, Moya-Sola S, Macchiarelli R (1999). *Oreopithecus* was a bipedal ape after all: evidence from the iliac cancellous architecture.. Proc Natl Acad Sci U S A.

[52] Moya-Sola S, Kohler M, Alba DM, Casanovas-Vilar I, Galindo J (2004). *Pierolapithecus catalaunicus*, a new Middle Miocene great ape from Spain.. Science.

[53] Tuttle RH, Ishida H, Tuttle RH, Pickford M, Ogihara N, Nakatsukasa M (2006). Are human beings apes, or are apes people too?. Human Origins and Environmental Backgrounds.

[54] Keith A (1923). Man's posture: its evolution and disorders. Hunterian Lectures I-VI.. Brit Med J.

[55] Tuttle RH (1974). Darwin's apes, dental apes, and the descent of Man: Normal science in evolutionary anthropology.. Curr Anthropol.

[56] Thorpe SKS, Holder RL, Crompton RH (2007). Origin of human bipedalism as an adaptation for locomotion on flexible branches.. Science.

[57] Ahlberg PE, Clack JA, Blom H (2005). The axial skeleton of the Devonian tetrapod *Ichthyostega*.. Nature.

[58] Springer MS, Murphy WJ, Eizirik E, O'Brien SJ (2003). Placental mammal diversification and the Cretaceous-Tertiary boundary.. Proc Natl Acad Sci U S A.

[59] Nilsson MA, Arnason U, Spencer PB, Janke A (2004). Marsupial relationships and a timeline for marsupial radiation in South Gondwana.. Gene.

[60] Douady CJ, Catzeflis F, Raman J, Springer MS, Stanhope MJ (2003). The Sahara as a vicariant agent, and the role of Miocene climatic events, in the diversification of the mammalian order Macroscelidea (elephant shrews).. Proc Natl Acad Sci U S A.

[61] Ishida H, Kunimatsu Y, Takano T, Nakano Y, Nakatsukasa M (2004). *Nacholapithecus* skeleton from the Middle Miocene of Kenya.. J Hum Evol.

[62] Ward CV (1993). Torso morphology and locomotion in *Proconsul nyanzae*.. Am J Phys Anthropol.

[63] Nakatsukasa M, Ward CV, Walker A, Teaford MF, Kunimatsu Y (2004). Tail loss in *Proconsul heseloni*.. J Hum Evol.

[64] Robertson JT (1972). Early Hominid Posture and Locomotion..

[65] Haeusler M, Martelli SA, Boeni T (2002). Vertebrae numbers of the early hominid lumbar spine.. J Hum Evol.

[66] Brunet M, Guy F, Pilbeam D, Mackaye HT, Likius A (2002). A new hominid from the Upper Miocene of Chad, Central Africa.. Nature.

[67] Guy F, Lieberman DE, Pilbeam D, de Leon MP, Likius A (2005). Morphological affinities of the *Sahelanthropus tchadensis* (Late Miocene hominid from Chad) cranium.. Proc Natl Acad Sci U S A.

[68] Zollikofer CP, Ponce de Leon MS, Lieberman DE, Guy F, Pilbeam D (2005). Virtual cranial reconstruction of *Sahelanthropus tchadensis*.. Nature.

[69] Wolpoff MH, Senut B, Pickford M, Hawks J (2002). Palaeoanthropology. *Sahelanthropus* or ‘*Sahelpithecus*’?. Nature.

[70] Harcourt-Smith WE, Aiello LC (2004). Fossils, feet and the evolution of human bipedal locomotion.. J Anat.

[71] Ohman JC, Lovejoy CO, White TD, Eckhardt RB, Galik K (2005). Questions about *Orrorin* femur.. Science.

[72] Galik K, Senut B, Pickford M, Gommery D, Treil J (2004). External and internal morphology of the BAR 1002'00 *Orrorin tugenensis* femur.. Science.

[73] Li G, Luo ZX (2006). A Cretaceous symmetrodont therian with some monotreme-like postcranial features.. Nature.

[74] Jenkins FA (1971). The Postcranial Skeleton of African Cynodonts; Problems in the Early Evolution of the Mammalian Postcranial Skeleton..

[75] Kielan-Jaworowska Z, Gambaryan PP (1994). Postcranial anatomy and habits of Asian multituberculate mammals. Fossils and Strata.

[76] Kielan-Jaworowska Z, Cifelli R, Luo Z-X (2004). Mammals from the Age of Dinosaurs: Origins, Evolution, and Structure..

[77] Kemp TS (2005). The Origin and Evolution of Mammals..

[78] Flower WH (1885). An Introduction to the Osteology of the Mammalia..

[79] Raaum RL, Sterner KN, Noviello CM, Stewart CB, Disotell TR (2005). Catarrhine primate divergence dates estimated from complete mitochondrial genomes: concordance with fossil and nuclear DNA evidence.. J Hum Evol.

[80] Williston SW, Gregory WK (1925). The Osteology of the Reptiles..

[81] Gregory WK (1947). The monotremes and the palimpsest theory.. Bull Am Mus Nat Hist.

[82] Hoffstetter R, Gasc J-P, Gans C, Bellairs AdA, Parsons TS (1969). Vertebrae and ribs of modern reptiles.. Biology of the Reptilia-Morphology A.

[83] Bezuidenhout AJ, Seegers CD (1996). The osteology of the African elephant (*Loxodonta africana*): vertebral column, ribs and sternum.. Onderstepoort J Vet Res.

[84] Slijper EJ (1946). Comparative biological-anatomical investigation on the vertebral column and spinal musculature of mammals.. Kon Ned Akad Wet Verh (Tweede Sec).

[85] Argot C (2003). Functional-adaptive anatomy of the axial skeleton of some extant marsupials and the paleobiology of the paleocene marsupials *Mayulestes ferox* and *Pucadelphys andinus*.. J Morphol.

[86] Filler AG (2007). The emergence and optimization of upright posture among hominiform hominoids and the evolutionary pathophysiology of back pain.. Neurosurgical Focus.

[87] Filler AG (2007). The Upright Ape: A New Origin of the Species..

[88] Fischer MS, Schilling N, Schmidt M, Haarhaus D, Witte H (2002). Basic limb kinematics of small therian mammals.. J Exp Biol.

[89] Schilling N, Hackert R (2006). Sagittal spine movements of small therian mammals during asymmetrical gaits.. J Exp Biol.

[90] Flynn JJ, Finarelli JA, Zehr S, Hsu J, Nedbal MA (2005). Molecular phylogeny of the carnivora (mammalia): assessing the impact of increased sampling on resolving enigmatic relationships.. Syst Biol.

[91] Keith A (1902). The extent to which the posterior segments of the body have been transmuted and suppressed in the evolution of man and allied primates.. J Anat Phys.

[92] Filler AG (1979). Functional and Evolutionary Perspectives on Chimpanzee Thoracolumbar Musculature..

[93] Owen R (1857). Osteological contributions to the natural history of the chimpanzees (*Troglodytes*) and orangs (*Pithecus*). No. V. Comparison of the lower jaw and vertebral column of the *Troglodytes gorilla*, *Troglodytes niger*, *Pithecus satyrus*, and different varieties of the human race.. Trans Zool Soc London, (printed 1862).

